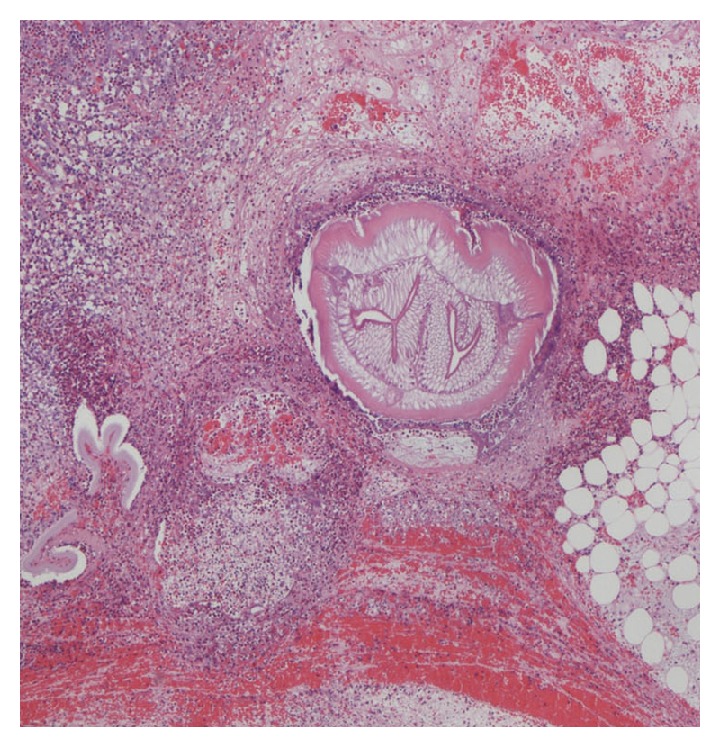# Common Symptoms from an Uncommon Infection: Gastrointestinal Anisakiasis

**DOI:** 10.1155/2016/5176502

**Published:** 2016-10-09

**Authors:** Yuto Shimamura, Niroshan Muwanwella, Sujievvan Chandran, Gabor Kandel, Norman Marcon

**Affiliations:** Division of Gastroenterology, St. Michael's Hospital, University of Toronto, 30 Bond Street, Toronto, ON, Canada M5B 1W8

## Abstract

Clinicians can be forgiven for thinking of anisakiasis as a rare condition low in the differential diagnosis of abdominal pain. Gastrointestinal anisakiasis is a zoonotic parasitic disease caused by consumption of raw or undercooked seafood infected with nematodes of the genus* Anisakis*. Even though the reported cases indicate that this is a rare disease, the true incidence of the disease could be potentially higher than what is reported in the literature as cases can go undiagnosed. Diagnosis and treatment of gastric anisakiasis are made by a compatible dietary history, direct visualization, and removal of the larvae via gastroscopy. Serologic testing and imaging studies are useful in the diagnosis of intestinal anisakiasis and conservative management should be considered. This disease may mimic other diseases and lead to unnecessary surgery. This emphasizes the importance of suspecting gastrointestinal anisakiasis by history taking and by other diagnostic modalities.

## 1. Introduction

Gastrointestinal anisakiasis is a zoonotic parasitic disease caused by consumption of raw or undercooked seafood infected with nematodes of the genus* Anisakis*. Those patients identified are predominantly infected by* Anisakis simplex *[1, 2] which is nematode belonging to the order Ascaridida, family Anisakidae, and subfamily Anisakinae [3, 4]. Only a handful of reports exist on infection related to the other species including* Pseudoterranova decipiens*,* Anisakis physeteris,* and* Contracaecum* species [2]. Marine mammals including whales, sea lions, seals, dolphins, porpoises, and walruses are the natural hosts whereas humans are incidental hosts [3] and cannot develop into adult nematode in the human body. The life cycle starts when adult nematodes in the natural host pass unembryonated eggs in their feces [5]. The eggs are embryonated, and then first and second stage larvae are formed within these eggs subsequently releasing free-living second stage larvae. These are ingested by crustaceans (intermediate hosts), in which they develop into the third stage. These can be passed on to fish and squid at their third stage larvae, which become infectious to humans when accidentally ingested [5, 6]. Salmon, herring, cod, mackerel, and squid are also well known intermediate hosts infected with the third stage larvae.

van Thiel et al. reported the first case of anisakiasis in 1960 [7]. It is commonly reported in coastal areas of Japan and Korea due to food habits. The increasing popularity of ingesting raw fish in Western countries has led to an increase in the number of clinical reports of anisakiasis [8–11] and there are some reports from North America including Canada [1, 12–14]. Vaughan et al. recently reported a case of a 50-year-old in Alberta, Canada, who presented with vomiting and epigastric pain one hour after eating raw salmon. It was diagnosed as gastric anisakiasis with esophagogastroduodenoscopy (EGD) and treated by removing the larvae [15]. There was a report on intestinal anisakiasis from Quebec, Canada, in 2003 [16]. A 50-year-old male from Quebec presented with abdominal pain after eating raw wild-caught salmon from the Pacific Ocean off Canada. Abdominal computed tomography (CT) scan showed bowel distention proximal to segmental wall thickening which was resected and* Anisakis* larvae were confirmed postoperatively. Given the increasing incidence in North America, it is important for clinicians in the appropriate context to consider anisakiasis as a differential diagnosis for patients presenting with nonspecific abdominal symptoms. We present two cases of anisakiasis that have recently been managed at our institution and summarize the available literature on the epidemiology, presenting symptoms and subsequent management.

## 2. Case 1

**Figure 1:**
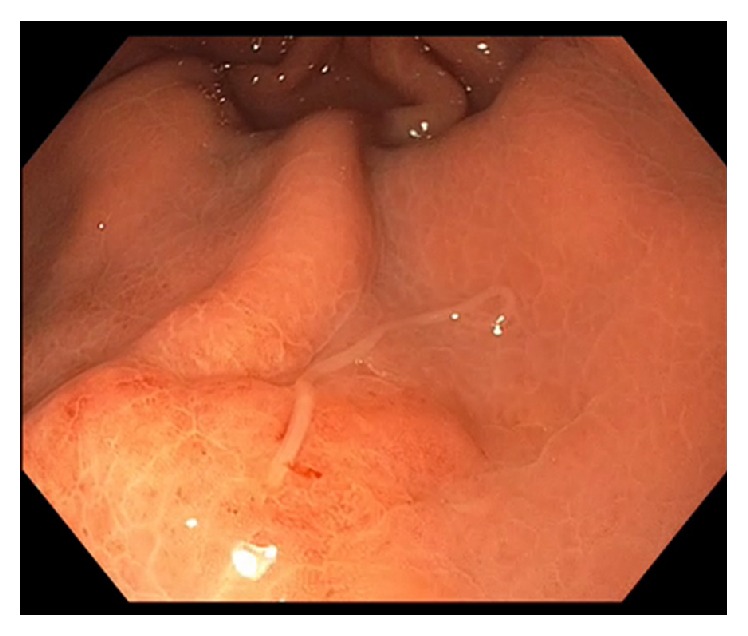


**Figure 2:**
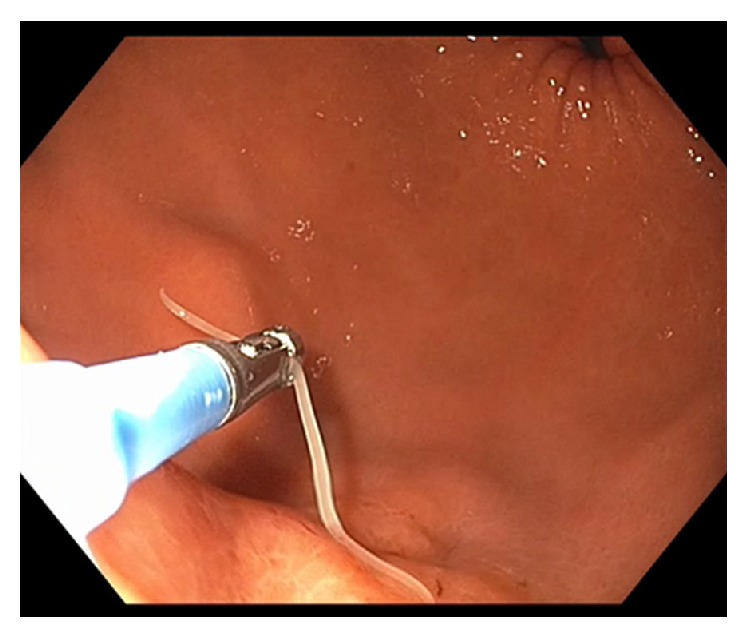


A 60-year-old Caucasian female was referred to our unit with a history of epigastric pain following sushi consumption. A CT scan showed thickened gastric mucosa in the distal body. EGD showed an area of mucosal induration and erythema with a parasite attached to this site (Figure 1). This parasite was carefully removed intact with the use of standard biopsy forceps (Figure 2) and microbiological examination confirmed as* Anisakis simplex*. The patient's clinical symptoms improved promptly following the endoscopic removal of the parasite [17].

## 3. Case 2

**Figure 3:**
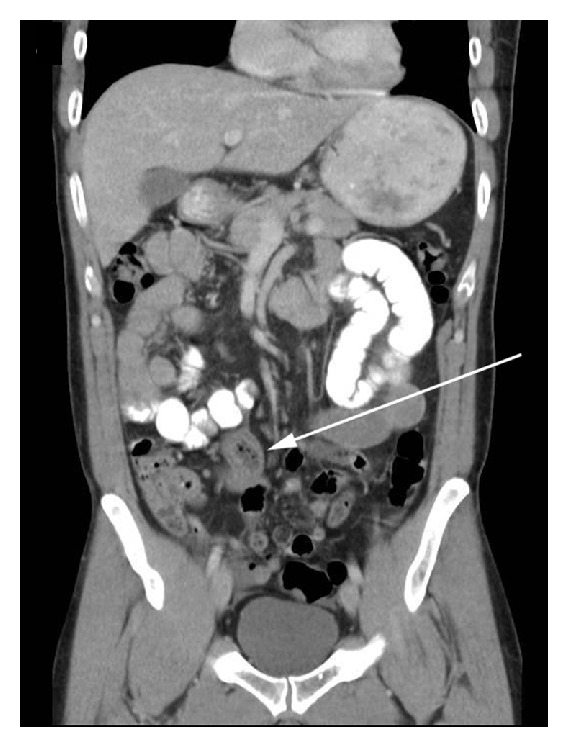


**Figure 4:**
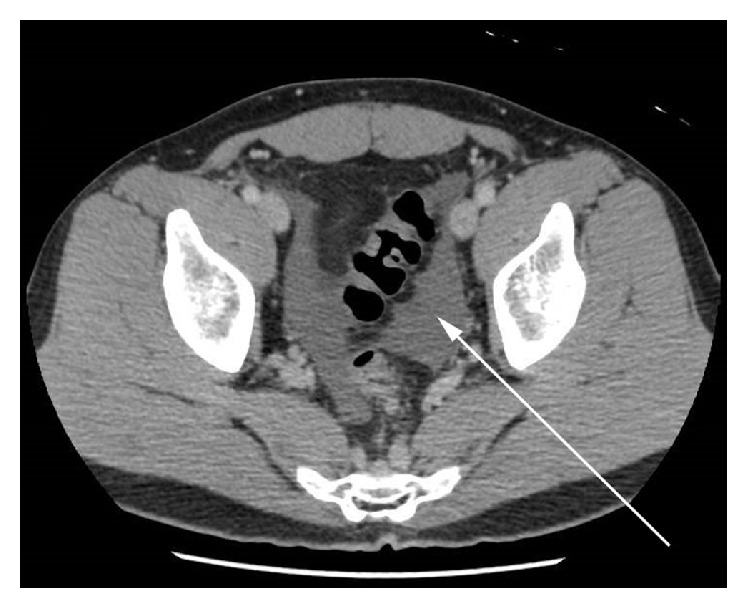


A 42-year-old Asian male presented to the emergency department with acute onset of colicky abdominal pain few days after eating sushi. He had a surgical abdomen on physical examination. He had an elevated white cell count and a CT scan obtained in the emergency revealed a segmental area of mural thickening in the proximal ileum and ascites (Figures 3 and 4). Given the surgical abdomen, he underwent exploratory laparotomy and a small bowel resection was carried out with primary anastomosis. Surgical specimen revealed extensive inflammatory infiltrate containing numerous eosinophils and lymphocytes extending from the mucosa deeply into the mesenteric adipose tissue. A larva was found embedded within the adipose tissue and was identified as* Anisakis* species (Figure 5) [18].

## 4. Gastrointestinal Anisakiasis

This disease can be divided into three categories: gastric, intestinal, and ectopic anisakiasis [19]. The majority of cases are gastric anisakiasis, representing about 95% of the disease burden. Intestinal anisakiasis accounts for the majority of the remaining [20] whereas the ectopic subtype or extragastrointestinal anisakiasis is a rare entity [21–25]. Interestingly, there are increasing reports of colonic anisakiasis which are mostly incidental findings [25–35]. Clinical manifestations not only are confined to gastrointestinal symptoms but also can cause allergic symptoms including angioedema, urticaria, and anaphylaxis [36]. Anisakiasis can be caused when the infected larvae are attached or penetrate into the human tissue. The etiology of this disease is not fully elucidated but it has been proposed that infection with* Anisakis* results in allergic host immune responses [3]. It is known that* Anisakis* predominantly induces the production of Th2 cytokines and subsequently causing mastocytosis, IgE mediated reactions, and eosinophilia which are classical immune response to tissue parasitic helminths [5, 37]. Consider that the extent of tissue destruction and inflammation resulting from infection indicates that the host-parasite interactions are responsible for the etiology of anisakiasis [6].

## 5. Clinical Manifestations

### 5.1. Gastric Anisakiasis

Gastric anisakiasis can be suspected based on the typical presentation, which is an acute severe epigastric pain few hours after the ingestion of infected fish. The symptoms usually develop within 12 hours [38, 39]. Other clinical manifestations include nausea, vomiting, and low-grade fever. There are cases in which the patients present with hematemesis from gastric ulceration [39–44]. There are asymptomatic cases identified incidentally. Interestingly, it tends to penetrate into normal mucosa more frequently than atrophic mucosa and patients with normal mucosa infection are more likely to exhibit clinical symptoms than those with atrophic mucosa [45, 46].

### 5.2. Intestinal Anisakiasis

The clinical characteristics are nonspecific but mostly present with colicky or diffuse abdominal pain, nausea, and vomiting. The symptoms typically develop within 5 days after the ingestion of infected food. It takes a longer time for symptoms to manifest compared to gastric anisakiasis [38]. The patients are often misdiagnosed with other diseases such as acute appendicitis, ileitis, diverticulitis, cholecystitis, inflammatory bowel disease, peptic ulcer, or small bowel obstruction. Intussusception is another rare presentation previously reported [47, 48]. The manifestation of this disease can occur a few days after ingestion, making the diagnosis challenging especially given the difficulties obtaining a history of raw fish consumption as the patient would often not remember what they ate several days prior to the presentation. According to Yasunaga et al., among 201 cases of intestinal anisakiasis identified in the Japanese Diagnosis Procedure Combination (DPC) in-patient database, 50.7% had bowel obstruction, 8.0% had perforation or peritonitis, and 2.0% had intestinal bleeding. Allergic responses were seen in 3.5% of the patients and 7.0% cases underwent laparotomy [49].

## 6. Diagnosis 

### 6.1. Gastric Anisakiasis

The diagnosis of gastric anisakiasis can be assisted by thorough history taking to identify consumption of raw fish. It is diagnosed by direct visualization of the larvae via EGD. The most frequent gastric mucosal change observed endoscopically is prominent gastric mucosal edema around the area of penetration [39, 50].* Anisakis* larvae seem to have predilection of penetrating the greater curvature of the stomach [50]. Narrow band imaging (NBI) may be helpful in detecting the larvae when performing an EGD [51]. Laboratory examinations such as elevated white cell counts with increased eosinophils may be helpful but leukocytosis with eosinophilia is infrequently seen according to case series from Korea [52]. Abdominal CT is useful to rule out any other causes of severe abdominal pain and the most frequent finding related to gastric anisakiasis is marked submucosal edema of the gastric wall. Increased attenuation of adjacent fat and ascites are other CT findings of this disease [53, 54]. Literatures which exist discuss the utility of abdominal ultrasound; however there is no better diagnostic modality than direct endoscopic visualization.

### 6.2. Intestinal Anisakiasis

The definitive diagnosis of the intestinal anisakiasis is often challenging, as direct identification of the nematode from small intestine is often not feasible. The most important diagnostic criteria are clinical features compatible with intestinal anisakiasis and history of ingesting raw or undercooked fish. Radiological findings especially abdominal CT are indispensable for diagnosis. Typical CT findings are segmental edema of the intestinal wall with proximal dilatation without showing complete intraluminal occlusion, ascites, and increased attenuation of adjacent fat [53, 55, 56]. Although abdominal ultrasound was inferior to CT in demonstrating the segmental intestinal edema causing small bowel obstruction, it can be applied in suspected cases especially when CT is not available [57, 58]. Another diagnostic modality is serology. Although there are reports on the usefulness of serology [1], it is not definitive. Anti-*Anisakis* IgG/A titers using an enzyme-linked immunosorbent assay (ELISA) are commercially available and considered useful with 70.4% sensitivity and 87.1% specificity [59–61]. The diagnosis may be supported by elevated total and* Anisakis* specific immunoglobulin E levels or to perform a prick test with crude parasite extract. However many asymptomatic subjects who frequently consume raw fish may also carry the specific IgE making the definitive diagnosis challenging [62–67]. In addition, Sastre et al. showed that the subjects who have shown hypersensitivity to* Anisakis simplex* did not experience clinical symptoms when they ingested lyophilized* Anisakis simplex* or its antigen [68]. Therefore it is hypothesized that the live larva secretes proteins that induce allergic type reactions in subjects and induce significant clinical symptoms. In addition, it is difficult to interpret since* Anisakis* proteins demonstrate considerable immunological cross-reactivity to proteins of related nematodes [3, 66, 69, 70]. Routine laboratory examination including leukocytosis, C-reactive protein, and peripheral eosinophilia may be helpful but these are not specific to this disease [38, 71]. Takabayashi et al. and Kim et al. reported that the patients with intestinal anisakiasis have higher possibility of having elevated white blood cell counts and C-reactive protein compared to gastric anisakiasis [38, 52]. Polymerase chain reaction (PCR) is reported to be useful in the diagnosis but not widely available [72]. Even if these tests are commercially available, it takes too long for the results making them redundant in the clinical practice. Currently there are no definitive diagnostic criteria; however we suggest that the following four criteria may be useful in diagnosing intestinal anisakiasis:

1. Clinical features compatible with intestinal anisakiasis.
2. History of ingesting raw or undercooked fish within 2 weeks.
3. Elevated levels of* Anisakis* specific IgE or* Anisakis* IgA/IgG (commercially available serologic tests).
4. The presence of segmental intestinal edema and distended small bowel proximally on CT scan.

Therefore patients with suspected infection based on their clinical symptoms and history of presenting complaint should undergo further testing with serology and CT imaging.

## 7. Treatments

### 7.1. Gastric Anisakiasis

The mainstay of the treatment is an early endoscopic extraction. It can be extracted by using the conventional forceps. It is important to grab as close to the embedded part of the larvae as possible to assure that there is no remaining larva within the gastric wall. If not removed completely, it can cause chronic inflammation causing various gastrointestinal symptoms. Thorough examination of all parts of the stomach is crucial as there is a possibility of multiple infections [41, 73–75]. There seems to be a predilection for penetrating into the greater curvature of the stomach body [39, 45]. Due to the rare occurrence of this disease, inexperienced endoscopists may easily overlook larvae since it is challenging to identify these larvae, especially in the greater curvature because they are usually hidden between the edematous gastric folds or blend in with the gastric mucosa. Extraction of the larvae will usually result in prompt symptom resolution. There is no definitive medical therapy as of date. There is limited evidence which suggests that albendazole is an effective therapy [1, 76, 77]. The anthelmintics do not appear to be effective therapy. There is limited literature on alternative medical therapies which include peppermint essential oil,* Melaleuca alternifolia* essential oil,* Matricaria chamomilla* essential oil in animal models [78, 79], and wood creosote (Seirogan) [80]; however these are not established as a standard treatment of anisakiasis.

### 7.2. Intestinal Anisakiasis

Symptoms of intestinal anisakiasis can be severe, presenting as bowel obstruction, and can resemble other acute abdominal diseases resulting in surgical treatment [19, 81–85]. Although surgical treatment was first-line treatment previously, there are increasing reports on effectiveness of conservative therapy [86–89]. There is still no standard treatment for intestinal anisakiasis; however conservative therapy should be considered if the disease is strongly suspected. On the other hand, some reports have shown that when supportive therapy failed the patients predominantly underwent surgery. Thus, careful observation of the patient is vital whilst having a low threshold for surgical intervention if clinically deterioration occurs given that there is no proven pharmacological therapy.

## 8. Prevention

The most important step of prevention is to educate the public about the risk of this disease when eating raw fish. These products should be inspected visually to detect the presence of visible parasites. There are different regulations and guidelines for the assessment of the nematodes. The Food and Drug Administration recommends raw or semiraw consumption be blast frozen to −35°C or below for 15 hours or be regularly frozen to −20°C or below for 7 days. Study on the survival of* Anisakis simplex* in fresh arrowtooth flounder (*Atheresthes stomias*) showed that all larvae were killed by 96, 60, 12, and 9 h at temperatures of −15, −20, −30, and −40 degrees C, respectively [90]. This study showed that, by following the FDA guidelines, all the live larvae could be eliminated.

## 9. Conclusion

Gastrointestinal anisakiasis is a rare parasitic disease that can affect humans following consumption of raw or undercooked seafood. A detailed food history will often be the key to the diagnosis as symptoms usually arise shortly after ingestion of food contaminated with the parasite. Even though the reported cases indicate that this is a rare disease the true incidence of the disease could be potentially higher than what is reported in the literature as cases can go undiagnosed. Treatment of gastric anisakiasis is by simply removing the larvae with biopsy forceps, which can lead to prompt symptom resolution. With regard to intestinal anisakiasis, conservative management is possible given the fact that the* Anisakis* larvae cannot survive in the human body; however the presentation can be such that surgical management is indicated.

We propose diagnostic criteria for intestinal anisakiasis as follows:

1. History of ingesting raw or undercooked saltwater fish within 2 weeks.
2. Clinical features compatible with intestinal anisakiasis.
3. Elevated levels of* Anisakis* specific IgE or* Anisakis* IgA/IgG (if available).
4. The presence of segmental intestinal edema and distended small bowel proximally on CT.

Diagnosis of intestinal anisakiasis using molecular or serological approaches is warranted, as this disease may be misdiagnosed leading to unnecessary surgery. However currently there are no commercially available serological tests that can diagnose anisakiasis with a high degree of sensitivity and specificity. This emphasizes the importance of suspecting gastrointestinal anisakiasis by history taking and by other diagnostic modalities.

## Figures and Tables

**Figure 5:**